# First Data on Efficacy and Safety of Nintedanib in Patients with Idiopathic Pulmonary Fibrosis and Forced Vital Capacity of ≤50 % of Predicted Value

**DOI:** 10.1007/s00408-016-9912-1

**Published:** 2016-07-04

**Authors:** Wim A. Wuyts, Martin Kolb, Susanne Stowasser, Wibke Stansen, John T. Huggins, Ganesh Raghu

**Affiliations:** 1 Unit for Interstitial Lung Diseases, Department of Respiratory Medicine, University Hospitals Leuven, Herestraat 49, 3000 Leuven, Belgium; 2 McMaster University, 50 Charlton Ave East, Hamilton, ON L8N 4A6 Canada; 3 Boehringer Ingelheim Pharma GmbH & Co. KG, Binger Str. 173, 55216 Ingelheim Am Rhein, Germany; 4 Medical University of South Carolina, 96 Jonathan Lucas Street, Charleston, SC 29425 USA; 5 University of Washington, 1959 N.E. Pacific, Campus Box 356175, Seattle, WA 98195-6522 USA

**Keywords:** Interstitial lung diseases, Clinical trial, Disease progression, Tyrosine kinase

## Abstract

In the Phase III INPULSIS^®^ trials, 52 weeks’ treatment with nintedanib reduced decline in forced vital capacity (FVC) versus placebo in patients with idiopathic pulmonary fibrosis (IPF). Patients who completed the INPULSIS^®^ trials could receive nintedanib in an open-label extension trial (INPULSIS^®^-ON). Patients with FVC <50 % predicted were excluded from INPULSIS^®^, but could participate in INPULSIS^®^-ON. In patients with baseline FVC ≤50 % and >50 % predicted at the start of INPULSIS^®^-ON, the absolute mean change in FVC from baseline to week 48 of INPULSIS^®^-ON was −62.3 and −87.9 mL, respectively (n = 24 and n = 558, respectively). No new safety signals were identified in INPULSIS^®^-ON compared with INPULSIS^®^. The decline in FVC in INPULSIS^®^-ON in both subgroups by baseline FVC % predicted was similar to that in INPULSIS^®^, suggesting that nintedanib may have a similar effect on disease progression in patients with advanced disease as in less advanced disease.

## Introduction

Idiopathic pulmonary fibrosis (IPF) is a chronic form of interstitial pneumonia associated with worsening dyspnoea and progressive decline in lung function [1]. Measuring decline in forced vital capacity (FVC) is an established method for assessing disease progression in patients with IPF and is a predictor of mortality [2, 3].

Nintedanib, an intracellular inhibitor of tyrosine kinases [4, 5], is approved for the treatment of IPF in several countries and regions, including the US, Europe [6, 7] and Japan. In the most recent international clinical practice guideline, nintedanib received a conditional recommendation for use in the treatment of IPF, taking into account individual patients’ preferences [8].

The INPULSIS^®^ trials were two replicate, randomised, placebo-controlled trials that assessed the efficacy and safety of 52 weeks’ treatment with nintedanib 150 mg twice daily (bid) in patients with IPF [9]. To be eligible to enter the INPULSIS^®^ trials, patients had to be ≥40 years old and to have an FVC of ≥50 % predicted, diffusing capacity of the lung for carbon monoxide of 30–79 % predicted and forced expiratory volume in 1 s/FVC ratio of ≥0.7. In both trials, nintedanib slowed disease progression by reducing the annual rate of decline in FVC by approximately 50 % [9]. Diarrhoea was the most frequent adverse event in the nintedanib group, reported in 62.4 % of patients treated with nintedanib and 18.4 % on placebo [9].

Patients who completed the INPULSIS^®^ trials were eligible to enter an open-label extension trial known as INPULSIS^®^-ON (NCT01619085), irrespective of their FVC. Therefore, patients with FVC ≤50 % predicted could participate in INPULSIS^®^-ON. We assessed the decline in FVC and safety of nintedanib in INPULSIS^®^-ON in patients who started this open-label extension trial with FVC ≤50 % and >50 % predicted.

## Methods

All patients who completed the 52-week treatment period and follow-up visit in an INPULSIS^®^ trial were eligible to enter INPULSIS^®^-ON. All patients provided informed consent before entering INPULSIS^®^-ON. Per protocol, the off-treatment period between the end of INPULSIS^®^ and start of INPULSIS^®^-ON could be 4–12 weeks. All patients in INPULSIS^®^-ON received open-label nintedanib. Patients who were receiving of nintedanib 150 mg bid or its matching placebo at the end of INPULSIS^®^ received of nintedanib 150 mg bid in INPULSIS^®^-ON, while patients receiving nintedanib 100 mg bid or its matching placebo at the end of INPULSIS^®^ could receive either nintedanib 100 or 150 mg bid in INPULSIS^®^-ON. The dose was decided based on discussion between the patient and investigator. As in the INPULSIS^®^ trials, dose reduction to 100 mg bid or treatment interruption was allowed to manage adverse events [9]. The primary outcome was the incidence of adverse events. Change in FVC was a secondary outcome measure. FVC measurements were taken at the start of INPULSIS^®^-ON, at weeks 2, 4, 6, 12, 24, 36, 48, 64, every 16 weeks thereafter until the end-of-treatment visit, and at a follow-up visit planned for 28 days after the last intake of the study drug.

The first patient was enrolled into INPULSIS^®^-ON in July 2012. The trial is ongoing. We conducted a post hoc, exploratory subgroup analysis in patients with FVC ≤50 % and >50 % predicted at the start of INPULSIS^®^-ON based on an interim database lock in November 2014. The analyses were performed for all patients treated in INPULSIS^®^-ON, irrespective of the treatment they had received in INPULSIS^®^. Absolute and relative declines in FVC in INPULSIS^®^-ON were based on observed cases. Safety was assessed via reporting of adverse events according to the Medical Dictionary for Regulatory Activities (MedDRA). All analyses in INPULSIS^®^-ON were descriptive.

## Results

A total of 1061 patients were treated in the INPULSIS^®^ trials (638 with nintedanib, 423 with placebo) [9]. Of 807 patients who completed the INPULSIS^®^ trials, 734 (91 %) patients were treated in INPULSIS^®^-ON (430 continuing nintedanib, 304 initiating nintedanib). Baseline FVC  % predicted values were missing for three patients at the start of INPULSIS^®^-ON (one continuing nintedanib, two initiating nintedanib). At the start of INPULSIS^®^-ON, 41 patients had FVC ≤50 % predicted (23 continuing nintedanib, 18 initiating nintedanib) and 690 patients had FVC >50 % predicted (406 continuing nintedanib, 284 initiating nintedanib). Baseline characteristics at the start of INPULSIS^®^ and INPULSIS^®^-ON are presented in Table 1. Mean (SD) exposure in INPULSIS^®^-ON was 16.7 (7.0) months. Mean (SD) duration of exposure in patients with FVC ≤50 % and >50 % predicted at the start of INPULSIS^®^-ON was 12.1 (8.6) and 17.0 (6.8) months, respectively.

**Table 1:** Baseline characteristics at start of INPULSIS^®^ and INPULSIS^®^-ON trials

	INPULSIS^®^		INPULSIS-ON^®^	
	Nintedanib (*n* = 638)	Placebo (*n* = 423)	FVC ≤50 % predicted (*n* = 41)	FVC >50 % predicted (*n* = 690)
Age, years, mean (SD)	66.6 (8.1)	67.0 (7.9)	66.9 (8.3)	67.1 (7.8)
Male, n (%)	507 (79.5)	334 (79.0)	32 (78.0)	554 (80.3)
Race, n (%)				
White	360 (56.4)	248 (58.6)	29 (70.7)	401 (58.1)
Asian	194 (30.4)	128 (30.3)	8 (19.5)	207 (30.0)
Black	2 (0.3)	0 (0.0)	0 (0.0)	2 (0.3)
Missing^a^	82 (12.9)	47 (11.1)	4 (9.8)	80 (11.6)
Ex or current smoker, n (%)	464 (72.7)	301 (71.2)	26 (63.4)	501 (72.6)
Weight, kg, mean (SD)	79.2 (16.6)	78.6 (16.5)	78.8 (17.6)	78.2 (16.1)
Body mass index, kg/m^2^, mean (SD)	28.1 (4.6)	27.6 (4.6)	27.1 (5.3)	27.5 (4.4)
FVC, mL, mean (SD)	2714 (757)	2728 (810)	1602 (330)	2683 (790)
FVC, % predicted, mean (SD)	79.7 (17.6)	79.3 (18.2)	45.0 (4.6)	78.0 (17.9)
FEV_1_/FVC, mean (SD)^b^	81.7 (6.0)	81.7 (5.8)	86.6 (7.4)	81.3 (6.5)

Based on data collected at start of INPULSIS^®^ or INPULSIS^®^-ON

^a^In France, regulation did not permit the collection of data on race

^b^n = 688 for subgroup of patients with FVC >50 % predicted at the start of INPULSIS^®^-ON

**Fig. 1:**
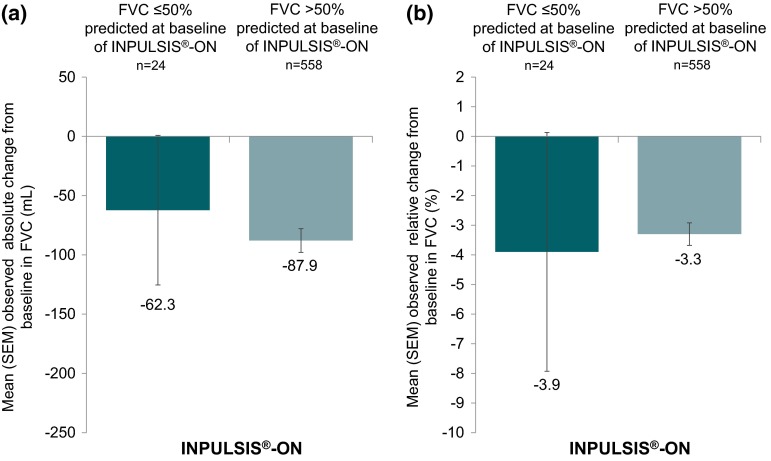
**a** absolute change in FVC from baseline to week 48 of INPULSIS^®^-ON, **b** relative change in FVC from baseline to week 48 of INPULSIS^®^-ON

FVC values from 24 patients with FVC ≤50 % predicted and 558 patients with FVC >50 % predicted at the start of INPULSIS^®^-ON were available at week 48. The absolute change in FVC from baseline of INPULSIS^®^-ON to week 48 of INPULSIS^®^-ON was of a similar magnitude in patients with FVC ≤50 % and >50 % predicted at the start of INPULSIS^®^-ON (mean [SEM] −62.3 [63.1] and −87.9 [10.0] mL, respectively) (Fig. 1a). Relative changes in FVC from baseline were also similar between subgroups (Fig. 1b). In both subgroups, the absolute change in FVC from baseline to week 48 of INPULSIS^®^-ON was of a similar magnitude to the absolute change from baseline of INPULSIS^®^ to week 52 of INPULSIS^®^ in patients treated with nintedanib in INPULSIS^®^ (mean [SEM] −88.9 [11.6] mL).

A summary of the adverse events in INPULSIS^®^ and INPULSIS^®^-ON is presented in Table 2. The most frequent adverse event in INPULSIS^®^-ON was diarrhoea, reported in 46.3 % and 64.6 % of patients with baseline FVC ≤50 % and >50 % predicted, respectively. Diarrhoea led to treatment discontinuation in 4.9 % and 5.4 % of patients in these subgroups, respectively. A higher proportion of patients with baseline FVC ≤50 % predicted compared with FVC >50 % predicted had adverse events of dyspnoea (24.4 % vs. 12.8 %) and progression of IPF (34.1 % vs. 15.1 %), which included disease worsening and acute exacerbations. Progression of IPF led to drug discontinuation in 17.1 % and 5.4 % of patients with baseline FVC ≤50 % and >50 % predicted, respectively. A higher proportion of patients with baseline FVC ≤50 % predicted than FVC >50 % predicted had serious adverse events (63.4 % vs. 39.3 %) and fatal adverse events (22.0 % vs. 9.6 %).

**Table 2:** Adverse events in INPULSIS^®^ and INPULSIS^®^-ON

	INPULSIS^®^		INPULSIS-ON^®^	
	Nintedanib (*n* = 638)	Placebo (*n* = 423)	FVC ≤50 % predicted (*n* = 41)	FVC >50 % predicted (*n* = 690)
Any adverse event(s)	609 (95.5)	379 (89.6)	41 (100.0)	649 (94.1)
Most frequent adverse event(s)^a^				
Diarrhoea	398 (62.4)	78 (18.4)	19 (46.3)	446 (64.6)
Nausea	156 (24.5)	28 (6.6)	7 (17.1)	111 (16.1)
Cough	85 (13.3)	57 (13.5)	7 (17.1)	114 (16.5)
Nasopharyngitis	87 (13.6)	68 (16.1)	3 (7.3)	100 (14.5)
Bronchitis	67 (10.5)	45 (10.6)	4 (9.8)	97 (14.1)
Dyspnoea	49 (7.7)	48 (11.3)	10 (24.4)	88 (12.8)
Progression of IPF^b^	64 (10.0)	61 (14.4)	14 (34.1)	104 (15.1)
Weight decreased	62 (9.7)	15 (3.5)	7 (17.1)	75 (10.9)
Severe adverse event(s)^c^	174 (27.3)	99 (23.4)	21 (51.2)	210 (30.4)
Serious adverse event(s)^d^	194 (30.4)	127 (30.0)	26 (63.4)	271 (39.3)
Fatal adverse event(s)	37 (5.8)	31 (7.3)	9 (22.0)	66 (9.6)
Adverse event(s) leading to treatment discontinuation^e^	123 (19.3)	55 (13.0)	17 (41.5)	155 (22.5)
Diarrhoea	28 (4.4)	1 (0.2)	2 (4.9)	37 (5.4)
Progression of IPF^b^	13 (2.0)	21 (5.0)	7 (17.1)	37 (5.4)
Nausea	13 (2.0)	0 (0.0)	1 (2.4)	5 (0.7)
Fatigue	1 (0.2)	1 (0.2)	1 (2.4)	3 (0.4)
Weight decreased	6 (0.9)	1 (0.2)	1 (2.4)	6 (0.9)
Decreased appetite	9 (1.4)	1 (0.2)	0 (0.0)	3 (0.4)

^a^Adverse events reported in >12 % of patients in either treatment group in INPULSIS^®^ and/or in the overall patient population in INPULSIS^®^-ON

^b^Corresponds to the MedDRA term ‘IPF’, which included disease worsening and acute exacerbations of IPF

^c^Events that were incapacitating or that caused an inability to work or to perform usual activities

^d^Events that resulted in death, were immediately life threatening, resulted in persistent or clinically significant disability or incapacity, required or prolonged hospitalisation, were related to a congenital anomaly or birth defect, or were deemed serious for any other reason

^e^Adverse events that led to permanent treatment discontinuation in ≥1 % of patients in the nintedanib or placebo group in INPULSIS^®^ and/or in the overall patient population in INPULSIS^®^-ON

## Discussion

In this interim analysis of data from INPULSIS^®^-ON, the open-label extension of the INPULSIS^®^ trials, the absolute decline in FVC over 48 weeks was similar in patients with FVC ≤50 % and >50 % predicted at the start of INPULSIS^®^-ON. In both subgroups, the decline in FVC in INPULSIS^®^-ON was similar to the decline in FVC in patients treated with nintedanib in the preceding INPULSIS^®^ trials. These results suggest that first, the effect of nintedanib on slowing disease progression is maintained beyond 52 weeks and, second, that patients with severely impaired FVC may receive the same benefit from nintedanib on reduction in FVC decline as patients with less severe impairment. In general, the adverse event profile was similar in both subgroups, with no new safety signals identified in INPULSIS^®^-ON compared to INPULSIS^®^, but as might be expected, serious adverse events and fatal adverse events were more common in patients with more advanced disease, as were adverse events of dyspnoea and disease progression.

These are the first data to be published on the effects of antifibrotic therapy in patients with FVC <50 % predicted. Patients with FVC <50 % predicted are usually excluded from clinical trials in IPF [9–13] as it is thought that it is too difficult to retain patients with severe lung function impairment in trials. However, studying this patient population is important, as patients with IPF and severely impaired FVC represent a patient group that require care in clinical practice [14]. Some physicians are already using antifibrotic drugs in patients with FVC <50 % predicted, based on regulatory authorities approving these drugs without restriction based on FVC [6, 7], but many physicians believe that the benefits of antifibrotic treatment would likely be reduced, and the side-effects worse, in patients with more advanced disease.

Subgroup analyses of pooled data from the INPULSIS^®^ trials have demonstrated a consistent effect of nintedanib on FVC decline in patients with baseline FVC ≤70 % versus >70 % predicted, ≤80 % versus >80 % predicted and ≤90 % versus >90 % predicted [15–17], suggesting that nintedanib is effective in reducing progression of IPF irrespective of the stage of disease at which it is initiated. The latest international clinical practice guideline for IPF highlights that there is no evidence to suggest an optimal duration of therapy or a point at which therapy would no longer be efficacious [8].

As with all data from open-label extension trials, these results have limitations, including bias in the population who completed the randomised placebo-controlled trials and elected to continue in the open-label extension and the lack of a placebo comparator in the extension phase. Findings from this subgroup analysis should be interpreted with caution as the number of patients with FVC ≤50 % predicted at the start of INPULSIS^®^-ON was small.

In conclusion, in an interim analysis of data from the INPULSIS^®^-ON open-label extension trial, the decline in FVC in patients with baseline FVC ≤50 % and >50 % predicted was similar to that in patients treated with nintedanib in INPULSIS^®^, suggesting a similar benefit on disease progression, and no new safety signals were identified.
